# A qualitative exploration of psychosocial determinants influencing viral load suppression among adolescents living with HIV: Application of social action theory

**DOI:** 10.4102/hsag.v31i0.3093

**Published:** 2026-01-26

**Authors:** Mathepe J. Thosago, Mygirl P. Lowane

**Affiliations:** 1 Department of Public Health, School of Health Care Sciences, Sefako Makgatho Health Sciences University, Pretoria, South Africa

**Keywords:** adolescents, antiretroviral therapy, human immunodeficiency virus, theory of social action, viral load suppression, South Africa

## Abstract

**Background:**

Adolescents living with HIV continue to experience poorer treatment outcomes than adults, with consistently lower rates of adherence and viral load (VL) suppression. This population faces unique developmental, social and emotional challenges that heighten their vulnerability.

**Aim:**

This study aimed to explore the psychosocial determinants influencing VL suppression among adolescents living with HIV (ALHIV) in Mpumalanga province, South Africa, by applying the Social Action Theory (SAT).

**Setting:**

The study was conducted at Mpumalanga province in South Africa.

**Method:**

An explorative, descriptive qualitative design was used, and data were collected from September to November 2023. Data were collected through in-depth interviews using a semi-structured interview guide in a local language. Nineteen participants were interviewed. Thematic analysis was employed, and the theory of social action; context influences, process of social and self-change and states of action were followed to orient the findings.

**Results:**

Six major themes, structured according to the SAT constructs, emerged, namely: Stigma and emotional distress hindering adherence and socio-environmental barriers to treatment consistency; Family and peer support promoting motivation for adherence and developing self-awareness and coping mechanisms; and lastly, Acceptance and responsibility towards treatment and formation of health-protective routines.

**Conclusion:**

Adolescents who understand HIV diagnosis and understand the benefit of antiretroviral treatment are actively engaged in targeted behaviour and are influenced by beliefs about their abilities and potential outcomes rather than passively driven by external forces.

**Contribution:**

There is a need for psychosocial support and monitoring from family members to help adolescents achieve the suppression of VL and to ensure adherence with antiretroviral therapy.

## Introduction

Human immunodeficiency virus remains a major public health concern globally, with the highest burden concentrated in Eastern and Southern Africa. In 2024, an estimated 40.8 million people were living with HIV worldwide, including 20.8 million in Eastern and Southern Africa, the region most affected by the epidemic (UNAIDS 2024; World Health Organization 2024). Although the rollout of Prevention of Mother-to-Child Transmission (PMTCT) programmes since 2004 significantly reduced vertical HIV transmission in South Africa and other countries, the number of adolescents living with perinatally acquired HIV has increased over time because of improved child survival following the expansion of paediatric Antiretroviral Therapy (ART) (Desmonde et al. 2021).

Globally, adolescents continue to experience poorer treatment outcomes compared with adults. UNICEF estimates that 1.57 million adolescents (10–19 years) were living with HIV in 2024, yet only 64% were receiving ART (UNICEF 2024). Among young people aged 15–24, ART coverage remained even lower at 59%, and viral suppression rates among adolescents varied widely, ranging from 44% to 88% across regions (Rakhmanina, Foster & Agwu 2024). Despite improvements in ART availability, adolescents persistently demonstrate lower adherence rates than other age groups, contributing to higher morbidity, viral resistance and mortality (Hlophe et al. 2022; Leshargie et al. 2022). Even in countries with strong ART programmes, such as South Africa, adolescents continue to report suboptimal treatment outcomes relative to adults (Foster, Ayers & Fidler 2020; Hlophe et al. 2025).

Adolescents living with HIV (ALHIV) face multiple challenges that contribute to these poorer outcomes. These include developmental transitions, emotional and mental health difficulties, unstable social support, and behavioural challenges linked to the pressures of adolescence (Kaseka et al. 2024; Mukuku, Govender & Wembonyama 2025). Cognitive and psychosocial maturation is still ongoing during adolescence, which can affect decision-making, self-regulation and the ability to maintain consistent adherence to long-term treatment. Mental health problems, including depression, anxiety and behavioural disorders, are particularly common among perinatally infected adolescents and can further undermine adherence (Olashore et al. 2021).

Psychosocial determinants such as stigma, family dynamics, mental health, peer relationships and healthcare interactions play a critical role in shaping adherence and treatment engagement in this age group. These determinants influence both the motivation and the ability of adolescents to maintain viral suppression. Although health-system interventions have focused on improving access to medication and HIV care (Audi et al. 2021; Elendu et al. 2025), emerging evidence shows that social relationships, emotional well-being and broader contextual factors are equally important in determining ART adherence and viral load (VL) outcomes (Ngwenya et al. 2024).

Despite increasing recognition of these challenges, there remains limited evidence on how psychosocial determinants specifically affect VL suppression among ALHIV. Understanding these influences is essential for developing interventions that address the unique needs of this population.

### Problem statement

Optimal VL suppression depends on consistent adherence to ART. However, evidence regarding the determinants of VL suppression among children and ALHIV remains limited (Mchomvu, Hussein & Matee 2022). Adolescents face unique challenges related to their developmental stage, family dynamics and social environment (Kaseka et al. 2024; Mukuku et al. 2025). Given that adherence behaviours are influenced not only by clinical factors but also by psychosocial determinants, such as stigma, emotional well-being, family support and peer influences, investigating these factors is essential to understand why VL suppression remains disproportionately low in this age group.

In South Africa, which has the world’s largest ART programme (Gandhi et al. 2025), adolescents continue to experience suboptimal HIV treatment outcomes (Haw et al. 2025). Between 2014 and 2020, VL suppression among children and adolescents aged 0–19 years showed minimal improvement compared to other age groups (Molopa et al. 2024). Longitudinal data from 2005 to 2019 indicate that VL suppression (< 400 copies/mL) was 72.4% among adolescents, compared to 85.7% among adults (Hermans et al. 2020; Pillay et al. 2020), highlighting their increased vulnerability.

Although interventions such as adherence clubs and counselling have been implemented, the relationship between these strategies and VL suppression remains unclear (Chamanga et al. 2024; Nkosi, 2025). Moreover, adolescents who struggle with disclosure, stigma and psychosocial stressors face additional barriers to adherence (Belzer et al. 2024; Hlophe et al. 2022; Liang, Wilson-Barthes & Galárraga 2024; Zaçe et al. 2024), underscoring the need to explore psychosocial determinants of VL suppression among ALHIV.

### Study purpose

The main purpose of this study was to explore, through a qualitative approach, the psychosocial determinants that influence VL suppression among ALHIV. The study was guided by the Social Action Theory (SAT) to better understand how individual, social and environmental factors interact to affect treatment outcomes.

## Research methods and design

### Study design

A descriptive qualitative design was followed to provide a contextual understanding of the subjective determinants affecting VL suppression among ALHIV and on ART. Using this research design not only helped to collect rich data on the phenomenon under study but also elicited valuable information about the phenomenon from the participants (Ranney et al. 2015).

**FIGURE 1:**
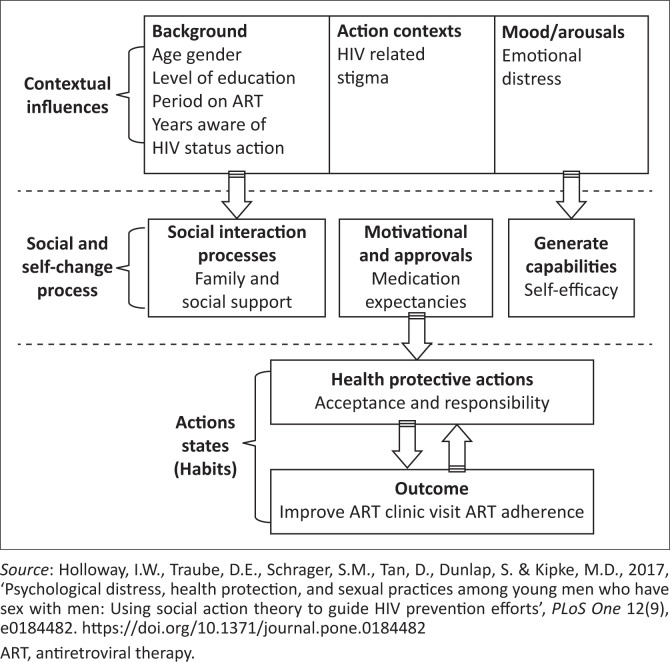
Application of social action theory to improve viral load among adolescents living with HIV.

The study used the theory of social action (SAT) to describe the motivations and meanings of adolescents associated with their experiences and actions, which are important to constantly promote the suppression of VL. The theory implies that all human actions or social actions are informed by the unique experiences, desires and contexts in which every human interaction occurs (Crosby & Di Clemente 2018). There is no fixed pattern of behaviour that everyone enacts. On the contrary, when individuals interact with people and the world around them and with various power systems, their behaviour changes accordingly. The SAT is a theory of behaviour change that considers the importance of the context in which health behaviour occurs, the process of self-regulation and social interaction driven by development, and the mechanisms by which these variables lead to behaviour that promotes health. The SAT also claims that health outcomes are a result of the interaction of three areas: context influence, self-changing process, health action and outcomes (Figure 1).

Many studies have focused on young people and adults living with HIV using SAT to identify factors associated with HIV transmission, risk behaviour, disclosure and treatment compliance. However, this study was informed by the SAT which serves as a comprehensive model for understanding health-related behaviours and outcomes by integrating personal, social and environmental influences through three interrelated domains: contextual influences, self-regulatory processes and social interactions. In this study, SAT was applied to examine how adolescents’ experiences in family, peer and healthcare contexts impact their adherence to ART and VL suppression. The theory was used to guide data collection, and the interview guide was designed to include questions that reflected each domain of the framework, such as contextual influences (e.g. stigma, family, health services), self-regulatory processes (e.g. motivation, coping, self-efficacy) and social interactions.

Moreover, the study was able to identify psychosocial determinants influencing VL suppression because the SAT domains guided the organisation and interpretation of themes during analysis. As a result, the theory was crucial for both formulating the research questions and understanding how adolescents deal with social and personal obstacles to improved ART clinic attendance and adherence so that VL suppression can be achieved.

### Study setting and population

The study was conducted in Mpumalanga province, South Africa. Mpumalanga is the second-smallest province in South Africa after Gauteng province. According to a report by the Human Sciences Research Council (HSRC 2024), the prevalence of HIV in Mpumalanga province was 17.4% across all age groups in 2022. Among adolescents and youth aged 15–24, the prevalence was 7.8% where 9.8% were male and 6.3% were female. In 2022, the percentage of ALHIV who were not virally suppressed was 73.0%, meaning that approximately one in four of them were not virologically suppressed (HSRC 2024). However, the available sources do not clearly report incidence data for adolescents in Mpumalanga, and more detailed age-band breakdowns of VL suppression among adolescents aged 10–19 years are also lacking. The study was conducted in five public primary healthcare facilities that offer comprehensive preventative, treatment care and rehabilitative services for HIV, Tuberculosis (TB) and Sexual transmitted infections (STIs).

The study population planned to target both female and male adolescents aged 10 years – 18 years living with HIV residing in Mpumalanga province; however, this study managed to access participants aged 14–18 years.

Adolescents initiated on ART for a period of 12 months with two latest consecutive unsuppressed VL results and knowing their HIV status were enrolled in this study. To avoid the chance of unintended disclosure of HIV, the study did not include adolescents who did not know their HIV status.

### Sampling and recruitment

The five healthcare facilities within the province with a high number of ALHIV on ART and those with unsuppressed VLs were purposively selected. The eligible participants were identified from the Three Interlinked Electronic Registers.Net (TIER.Net), after determining the population of adolescents living with HIV clients enrolled on ART from all those facilities in the study setting. TIER.Net is primarily used to extract and monitor routine data for clinical care and treatment of HIV patients in public health facilities in South Africa (Govere et al. 2023).

Purposive sampling was used to select adolescents with unsuppressed VL during data collection. The principal investigator then wrote the participants’ names who met the eligibility criteria of this study and placed them in a bowl, and asked a data capture to select six candidates. Because of the sensitivity of the study, the facility healthcare worker working with ART services contacted the selected participants for their availability to participate in the study and to ensure confidentiality. If the selected candidate was unavailable, not reached or declined to participate, the next person would be selected to maintain the number of six candidates per facility to certify the rule of thumb for qualitative study. Sharma et al. (2024) mentioned that rules of thumb are based on methodological considerations and prior experience, but they lack a clear and comprehensive justification for sample size. For qualitative research designs, several researchers have suggested general guidelines for sample size. These general guidelines suggest a sample size of 4 to 30 for single case studies and 5 to 35 for grounded theory (Sharma et al. 2024). For this study, a target of 30 participants was set. In descriptive qualitative research, a sample of this size is generally considered sufficient to achieve data saturation while capturing diverse perspectives (Sharma et al. 2024). The primary aim was to balance the depth of individual experiences with the breadth of viewpoints necessary to produce comprehensive and meaningful findings.

### Data collection

The facility healthcare worker again introduced the purpose of the researcher’s visit to the adolescents who agreed to participate. After that, the researcher took over by making an appointment and arranging with the participants who had agreed. Data were collected at the clinic in a room provided by the operational manager after the participant collected the medication and other routine check-ups. An in-depth semi-structured interview guide addressing the SAT constructs was used during data collection. Before commencing with the actual data collection sessions, the interview guide was pretested before the main study with two participants in another facility. The principal investigator conducted the individual interviews in the language of the interviewee’s preference, in English, isiZulu or SiSwati. The interviews revolved around the following questions, which were followed by more in-depth probing to expand the richness of information gathered:

- *Can you tell me about your experience of taking your HIV medication every day?*
- *What makes it difficult for you to take your medication sometimes?*
- *What helps you to take your medication on time?*
- *What do you think are the reasons some adolescents struggle to keep their viral load low?*

Informed consents and permission to record the interviews were obtained. Each interview lasted from 40 to 45 min on average, and some went beyond because of what transpired during the discussion. Interviews were conducted in a noise-free consulting room at the healthcare facility allocated by the facility manager. A total of 19 participants were interviewed, and data saturation was declared at the 19^th^ interview. An audio recorder was used throughout the data collection period and sessions. Field notes were also taken to support the interviews. Data were collected from September to November 2023.

### Data analysis

A deductive thematic analysis linked to Braun and Clarke’s (2006) six-phase framework was conducted. This approach was chosen because it is flexible, organic and emergent through the coding process. Using thematic analysis assists with conducting exploratory studies when a small amount of a particular phenomenon is known (Vaismoradi, Turunen & Bondas 2013). Data collected were transcribed verbatim, and the transcripts were translated into English, where interviews were in isiZulu, by the researcher and confirmed by the supervisor. The researcher read the transcript several times to become familiar with the transcribed data, which assisted with developing codes. Transcribed data were coded and labelled, and a codebook was created to indicate the consistency of the codes or labels used. NVivo software was used to analyse and transfer transcribed data.

Themes were generated in line with the SAT constructs (see Table 2).

### Trustworthiness and bias

Measures to ensure trustworthiness were followed for the systematic rigour of the research design. Four general criteria in the approach to trustworthiness are credibility, transferability, dependability and confirmability, according to Lincoln and Guba (1985), were followed. The study achieved credibility by applying prolonged engagement and time spent with the participants during data collection until data saturation occurred. Transferability was ensured through providing an audit trail of the research process. To ensure dependability, IDIs were conducted, and a quality audio recorder and field notes were used to allow for the accurate transcription of the transcript. The authors ensured that the findings were based on the data collected from the participants. An audit trail was maintained throughout data collection, analysis, and interpretation to enhance confirmability.

Recall bias was minimised by avoiding questions that required participants to recall distant past events. Instead, questions focused on current experiences with adherence and healthcare engagement. Because HIV is a sensitive topic, there remains a possibility of social desirability bias, where participants may respond in ways they believe are acceptable to the interviewer. Purposive sampling ensured that participants were selected based on their experiences and relevance to the research question, enhancing depth rather than generalisability.

### Ethical consideration

Ethical clearance to conduct the study was obtained from Sefako Makgatho University Research and Ethics Committee (SMUREC/H/111/2022: PG) and permission to conduct the study was sought from the Mpumalanga Provincial Department of Health (DoH) and from operational managers in selected facilities. Parents or caregivers gave consent for the minor adolescents to participate in this study and the participants less than 18 years of age, signed the assent form. Participants aged 18 years signed the informed consent forms. All the participants were informed about their right to withdraw from the study at any time if they did not wish to continue. Numerical codes were used during the interview to protect the identity of the participants. Confidentiality was maintained by keeping the signed consent and/or assent form in a safe area to which only the researchers had access.

## Results

### Socio-demographic characteristics

Eleven participants were female, while eight were male. Ten participants mentioned that they were living with their parents, while nine were living with relatives. The number of participants taking medication without the supervision of their parents or guardians was 13, and 6 were directly observed by their family members. Of the 19 participants, 6 were attending primary school, 11 secondary school and 2 passed grade 12 (Table 1).

**TABLE 1:** Summary of participants’ description.

Participant number	Age (years)	Gender	Living with	Supervisor of treatment	Educational level
1	14	Female	Parents	Parent	Grade 08
2	18	Male	Parents	Self	Passed grade 12
3	16	Female	Relative	Self	Grade 10
4	15	Male	Parents	Self	Grade 09
5	16	Male	Relative	Self	Grade 08
6	17	Male	Parents	Self	Grade 09
7	17	Male	Parents	Parent	Grade 09
8	14	Male	Parents	Self	Grade 06
9	14	Female	Parents	Parent	Grade 09
10	13	Female	Relative	Self	Grade 05
11	13	Female	Relative	Self	Grade 07
12	17	Female	Parents	Self	Grade 09
13	12	Female	Parents	Parent	Grade 04
14	14	Female	Relative	Self	Grade 08
15	13	Male	Relative	Parent	Grade 06
16	18	Female	Relative	Self	Passed grade 12
17	16	Female	Relative	Self	Grade 09
18	18	Male	Relatives	Self	Grade 10
19	14	Female	Parents	Parents	Grade 07

### Themes and sub-themes driven from social action theory constructs

A total of 6 major themes and 12 sub-themes emerged, structured according to the SAT constructs, namely Contextual Influences, Self-Change Processes and the Action States (Habits) (Table 2).

**TABLE 2:** Social action theory constructs, data-driven themes, and sub-themes.

SAT constructs	Themes	Sub-theme
1. Contextual Influences	1.1 Stigma and emotional distress hinder adherence	1.1.1 Fear of being judged and concealment of medication because of stigma 1.1.2 Emotional distress and anxiety leading to poor adherence
	1.2 Socio-environmental barriers to treatment inconsistency	1.2.1 Distance to health facilities 1.2.2 Inconsistent caregiver supervision
2. Self-Change Processes	2.1 Family and peer support promote motivation for adherence	2.1.1 Supportive care alongside limited disclosure about HIV status 2.1.2 Peer acceptance reducing feelings of isolation
	2.2 Developing self-awareness and coping mechanisms	2.2.1 Understanding the meaning of HIV diagnosis 2.2.2 Desire to live a healthy life despite diagnosis
3. Action States (Habits)	3.1 Acceptance and responsibility towards treatment	3.1.1 Taking responsibility for own medication and building self-discipline to treatment
	3.2 Formation of health-protective routines	3.2.1 Establishing medication routine 3.2.2 Recognising that adherence prevents illness and death 3.2.3 Routine follow-up in healthcare facilities

SAT, social action theory.

#### Theme 1.1: Stigma and emotional distress hinder adherence

Adolescents described experiencing strong feelings of shame, fear and emotional distress following their HIV diagnosis. Many avoided taking treatment in front of others to conceal their status. Fear of being judged or labelled resulted in inconsistent adherence and missed clinic visits.

**Sub-theme 1.1.1:** Fear of being judged and concealment of medication because of stigma: The biggest concern for adolescents was judgement and stigmatisation. Some expressed the fear of rejection from friends, family and community members. Others mentioned the question of judgement and rumours about their status:

‘People can’t always be trusted. If I tell my friends about my status, I don’t know what might happen next. I worry they’ll judge me and treat me differently or even tell everyone.’ (14-year-old female, Participant 14)

Some participants stated that they purposefully conceal their treatment by putting it in a different container because they are afraid of being judged if others find out they are HIV-positive. Others added that they avoid taking their antiretroviral medication in public, such as at school or in front of other family members. They consequently fail to take the doses or take them at the incorrect time:

‘To keep people from seeing my pills, I conceal them in a different container. This helps me occasionally because I can take them in public or with friends without raising any red flags. However, this isn’t always the case because it makes them wonder why I take headache medication daily. As a result, I occasionally forego them and take them later in the day.’ (14-year-old female, Participant 9)

**Sub-theme: 1.1.2:** Emotional distress and anxiety leading to poor adherence: Adolescents explain the emotional factors that cause non-adherence to ART, such as confusion, depression, guilt, suicidal thinking and overthinking. Some of the participants responded to emotional factors:

‘I don’t understand on why does it have to be me, what confuses me the most is my elder sister and the first born she is not on treatment, me being the second born and I am on treatment, and the last born is also not on treatment, why does have to be me? I would question myself that, why does it have to be me only who is living with HIV and not my other siblings. I sometimes wish to commit suicide.’ (14-year-old female, Participant 19)

‘I have not stopped taking the treatment, but I sometimes think of stopping the treatment. And I do not know why I am thinking like that. Sometimes I will be thinking too much, especially when they remind me about my appointment date in the facility, and I do not want to go to the facility.’ (16-year-old female, Participant 3)

#### Theme 1.2: Socio-environmental barriers to treatment inconsistency

Participants reported structural and environmental challenges, including long distances to clinics, a lack of privacy and inconsistent caregiver supervision. These barriers disrupted treatment routines and affected their ability to maintain VL suppression.

**Sub-theme 1.2.1:** Distance to health facilities: Participants explained how having to travel long distances between the clinic and school made it difficult for them to get antiretroviral therapy on time. Participants who reside in rural locations mentioned difficulties such as time lost from school, expensive transportation and even limited transportation options:

‘I cannot afford to go to school and the clinic at the same day. I don’t want anyone to suspect anything, more especially the teachers because they ask many questions about my whereabout. So, I prefer to attend school instead.’ (17-year-old male, Participant 6)

‘Transportation cost is a challenge. I skip going to the clinic because of not have enough money to return to school.’ (15-year-old male, Participant 4)

According to some participants, missing clinic appointments is because of the practical barriers that frequently led to treatment interruptions and delays in treatment refills, which in turn jeopardised the suppression of VL:

‘Sometimes I run out of pills because I miss my clinic days. I end up missing doses because I must wait until the next appointment. I find it challenging to maintain a low viral load as a result.’ (13-year-old female, Participant 12)

**Sub-theme 1.2.2:** Inconsistent caregiver supervision: Some participants mentioned that poor medication adherence was because of the inconsistent or insufficient caregiver support. Other participants emphasised that some caregivers or parents were unavailable because of work obligations or did not fully comprehend the significance of strict ART adherence to them. Some participants mentioned that their parents or caregivers showed little interest in keeping track of medication taking or clinic attendance. As a result, they frequently forgot doses or neglected to refill medication on time when caregiver supervision was inconsistent:

‘Even I forget to take the medication, and my aunt doesn’t give a damn. She doesn’t even remind me of the clinic date. No one is checking on me If I take my treatment as prescribed.’ (13-year-old male, Participant 15)

‘My mother simply leaves for work, arrives home late, and goes to bed. I occasionally remember to take the medication on my own, especially when I am in pain.’ (17-year-old male, Participant 6)

#### Theme 2.1: Family and peer support promote motivation for adherence

Social support is a potent adherence facilitator. Participants emphasised that reminders and support from caregivers/parents and friends increase their desire to take their medication regularly. Some participants stated that their parents accompany them to the clinic for follow-up appointments and constantly remind them to take the medication:

‘Usually, I don’t forget to take my medication. When Generation Soapy (on Television) begins, my mother always gives me the medication. She always calls to let me know where to take the medication if she is away.’ (14-year-old female, Participant 1)

**Sub-theme 2.1.1:** Supportive care alongside limited disclosure about HIV status: While conducting the interview, the participants explained the type of support received from family members after HIV diagnosis, the benefits of treatment and the associated risks:

‘My mother will tell me that she loves me and will continue to take care of me, even though she advised me not to tell anyone that I am living with HIV. She [*mother*] would tell me how a person living with HIV should live.’ (14-year-old female, Participant 9)

‘My mother told me that now that I am living with HIV, it doesn’t mean that I will die or anything of that sort, I must just take my treatment as being told, eat well, I won’t succumb to HIV.’ (18-year-old female, Participant 16)

In contrast, some participants cited a failure to achieve an undetectable VL because of not disclosing their HIV status. According to some participants, they were not informed of the reason behind their daily use of ARVs, so they do not bother to take them regularly. They were informed by their parents or guardians that they should take the medication for other illnesses:

‘My mother never told me that these pills are for HIV, but I took them daily, but I asked her why I am taking these pills daily. That is when she told me.’ (14-year-old female, Participant 1)

‘She simply told me that since she received treatment as a child, I should do the same without providing any justification.’ (13-year-old female, Participant 10)

‘I’ve been told that I have been taking that treatment since I was born, but I never got a clear explanation, so I’m not sure. She [*mother*] mentioned that I since after birth I was having trouble breathing.’ (16-year-old male, Participant 5)

**Sub-theme 2.1.2:** Peer acceptance reducing feelings of isolation: Participants identified peer acceptance as a key social factor that strengthened their reinforced adherence behaviour and enhanced their VL suppression. Some participants reported feeling less alone and socially isolated because their friend accepts them regardless of their HIV status. In addition, some participants stated that they felt more valuable and emotionally supported while they were in peer adherence clubs where HIV issues were discussed candidly:

‘Some of my friends go with me to the clinic to pick up the treatment, but otherwise they just treat me normally. I can see that they are also impacted when I’m feeling down. They will inquire as what’s wrong and whether they can offer help.’ (18-year-old male, Participant 18)

‘Yes, I do go to adherence clubs, and it’s beneficial. I’ve come to the conclusion that if a stick to my treatment, I won’t need to worry too much about my condition and believe that having HIV is not a curse; it was merely unfortunate.’ (18-year-old female, Participant 16)

#### Theme 2.2: Developing self-awareness and coping mechanisms

Participants showed greater dedication to treatment when they were aware of their HIV diagnosis and the goal of ART. Self-efficacy and resilience were promoted by knowledge of the advantages of treatment and a desire for a healthy future. Some reported that they stopped missing the pills once they realised how beneficial the medication was for them.

**Sub-theme 2.2.1:** Understanding the meaning of HIV diagnosis: Participants pointed out that their HIV status had led to different misunderstood feelings. They mentioned confusion about HIV diagnosis. Some participants reported being personally affected, while others described its impact on their school life or interactions with peers. Nevertheless, they felt comforted once they understood their HIV diagnosis:

‘The doctor explained to me, but when he told me that I am HIV positive, I could not hear anything more, because I was truly shocked. But when the doctor explained it in detail, I had to understand the situation. Although I am still confused, I am comfortable with the understanding why I should take the medicine for the rest of my life.’ (18-year-old male, Participant 2)

‘At school, we learned about it that people who are living with HIV are normal it just never got to me. But when I found out that I am positive, my mother told me that, there is nothing wrong with me, I am just living with a virus, if I am taking my treatment, I do not need to worry about anything.’ (13-year-old female, Participant 11)

‘I understand that living with HIV will kill you if you don’t take your medication, and it might progress to the point where it becomes AIDS.’ (15-year-old female, Participant 4)

**Sub-theme 2.2.2:** Desire to live a healthy life despite diagnosis: Despite their HIV diagnosis, nearly all participants demonstrated a strong determination to maintain their health and lead fulfilling lives. Many participants explained that continuing their treatment was a means of securing a future free from illness and pursuing personal objectives such as finishing school, starting a family and becoming independent. They also hinted that their adherence to ART was a major factor in their desire for longevity and normalcy because they knew that they would not be able to suppress their VL if they did not take their medication regularly:

‘I used to be ill and denied that I had HIV, but I came to terms with it and am now feeling better. I will make every effort to focus on taking the medication as prescribed rather than on my condition.’ (17-year-old female, Participant 7)

‘I feel better knowing that the rest of my life will remain this way. However, I will continue to go to school and attend a clinic every month. It is not important to me to continue to tell the teachers that I will pass the clinic to collect medicine on a particular day, which is why I cannot attend morning classes.’ (14-year-old female, Participant 14)

#### Theme 3.1: Acceptance and responsibility towards treatment

Many adolescents expressed that accepting their HIV status marked a turning point in adherence. Acceptance enabled them to take ownership of their treatment and view ART as part of their daily life.

**Sub-theme 3.1.1:** Taking responsibility for own medication and building self-discipline in treatment: Participants explained how they accepted their HIV status, started treatment, and learned how to live with their condition. Some participants were positive about treatment and were optimistic about the future, while others struggled to reconcile the attitudes when they arrived late at school after fetching medicines at the clinic and at their home:

‘There’s more to life; I’d prefer to receive treatment. When my alarm goes on at 20:00, I just accept it and take my medication; that’s all I do to maintain my health.’ (14-year-old male, Participant 8)

‘Now I know it’s my life. I must take care of myself.’ (13-year-old female, Participant 11)

#### Theme 3.2: Formation of health-protective routines

Regular clinic visits and the implementation of structured medication regimens were linked to consistent adherence. Regular adherence was associated by the participants with better health, fewer symptoms and a feeling of normalcy. Almost all said that they simply take their medications ahead of schedule and that it’s just a part of their morning or evening routine.

**Sub-theme 3.2.1:** Establishing medication routines: The adolescents described their treatment routines, reporting adherence to their medication schedules and using various types of reminders to ensure they took their medication consistently:

‘After eating, around seven. Because I was told to take them after meals. But now my father has given me time to take my treatment, which it is seven o’clock.’ (17-year-old male, Participant 6)

‘I take my medication daily after my meal, I quickly eat, so at around 8pm I can take my medication. Because I was told to take my medication at that immediate hour, I take them daily.’ (14-year-old female, Participant 1)

Some have stated that they use timers on their phones, watches or alarm clocks to remind them to take their medication as part of their daily routine. Others indicated that they take their medicine before or after evening meal, which is normally taken around the same time daily.

**Sub-theme 3.2.2:** Recognising that treatment adherence prevents illness and death: The adolescents explain the importance of adhering to the treatment. Most participants expressed that they did not want to be sick and wanted to protect themselves. Some participants pointed out:

‘I want to be better because I do not want to be back in the situation where I drink a lot of pills again. I do not want to see myself in that situation again.’ (18-year-old female, Participant 2)

Some participants expressed concern about the consequences of stopping treatment. They knew that it was important to take drugs at the right time every day because HIV would continue to spread in their body if they did not.

**Sub-theme 3.2.3:** Routine follow-up in healthcare facilities: Adolescents shared their reasons for visiting the facility and the emotional effects of facility visits. All participants mentioned that they visited the facility for treatment and VL monitoring:

‘I go there to collect the treatment, get the next appointment date and do blood tests.’ (17-year-old female, Participant 7)

Some adolescents initially struggled with attending clinic appointments, with some avoiding visits out of fear of being questioned while others frequently skipping appointments because of fatigue from routine visits. However, two participants came to recognise the importance of regular check-ups; understanding their condition and receiving support from nurses helped them to stay on track with their treatment and remain motivated to take their medication consistently:

‘I feared they would find something wrong or ask too many questions, so I avoided going to the clinic. But now I understand my condition, and nurses are there to help me not to become ill.’ (12-year-old female, Participant 13)

‘I was tired of regular appointments, so I used to skip a lot. Consequently, my viral load was always high. Nurses were always shouting at me with a concern. Now I make sure I don’t miss my clinic appointments, because I receive regular checkup to see if my treatment is working or not. This helps me to be on track with my medication and be motivated to take them.’ (17-year-old female, Participant 7)

## Discussion

In this study, the authors aimed to explore the psychosocial determinants influencing VL suppression among ALHIV on ART, using a modified SAT framework to examine how contextual influences, self-change processes, and action states shape adherence and treatment outcomes. The results of this study are consistent with evidence from around the world that indicates adolescents are still the least probable age group to achieve VL suppression, compared to adults (Rakhmanina et al. 2024).

In the theory of social action, contextual influences determine how the environment and social structures shape individual actions (Smelser & Baltes 2001). The contextual influences of adherence included stigma, emotional distress and socio-environmental barriers. Adolescents in this study reported persistent fears of stigmatisation and judgement, with many expressing concerns about being rejected by friends, family or community members. This aligns with broader conceptualisations of HIV-related stigma, which describe it as both a social process involving negative attitudes and behaviours towards people living with HIV and a psychological burden that shapes how individuals perceive themselves (Gruszczyńska & Rzeszutek 2023).

These fears influenced adolescents’ daily treatment behaviours, including concealing medication and feeling anxious when taking treatment in front of others – actions that reflect how stigma can interfere with self-regulation and adherence.

Similarly, studies conducted in Malawi and Eswatini have shown that stigma remains a significant predictor of viral non-suppression among adolescents living with HIV (Hlophe et al. 2025; Msefula & Umar 2023).

Together with the current findings, this suggests that HIV-related stigma continues to undermine treatment adherence and threaten optimal health outcomes for adolescents, despite improvements in HIV treatment and care.

Emotional state is related to distress and anxiety, and other mental health status of adolescents has a strong influence on VL suppression (Gordon et al. 2022). In this study, emotional factors that result in non-adherence to ART include confusion, depression, guilt, suicide and overthinking. Literature indicates that depression is reported to be a barrier to the suppression of VL (Huang et al. 2025). Several pathways, such as behavioural, cognitive, psychosocial and biological mechanisms, may explain how depression acts as an obstacle to the suppression of VL (Huang et al. 2025). Depression leads to a loss of interest in everything, including HIV-adherence. With compromised adherence to ART, achieving viral suppression becomes highly unlikely (Izudi et al. 2024).

This study suggests that adolescents do not follow treatment because of emotional factors, and different studies provide similar conclusions on the relationship between emotional status and viral suppression. For example, the study conducted in Botswana by Olashore et al. (2023) found that adolescents with high scores of cognitive, emotional and behavioural problems had higher unsuppressed VLs than those with lower scores.

Supported by the study of Haas et al. (2020), which provides convincing evidence of the correlation between mental health status and the suppression of viruses, Zhan et al. (2024) highlighted that ALHIV suffers from mental health complications, including depression, anxiety and traumatic stress disorders (PTSD). Mental, emotional and physical healthcare services should be provided in the healthcare facilities in which members of the enrolled adolescents club access ART (Msefula & Umar 2023).

The context of self-change suggests that individuals can model behaviour themselves by collective action and social interaction means that a person can act and react to those around them. In this study, the authors identified that the support of family members in relation to the disclosure was compromised. Some adolescents were not informed about their HIV-positive status but accidentally discovered their HIV status, and others were prohibited from disclosing their status. Evidence shows that adolescents who disclose their HIV status experience fewer challenges with ART adherence than those who do not (Hlophe et al. 2023; Mengesha et al. 2023).

Conversely, adolescents who have not disclosed their status are often compelled to behave ‘normally’ around family and peers, which leads them to hide their medication and avoid taking treatment in the presence of others (Audi et al. 2021; Nxumalo, Van Rensburg & Jacobs 2024; Smith et al. 2023). Such concealment can disrupt consistent adherence and increase the risk of viral non-suppression.

Parents who have not informed adolescents of their HIV status have difficulty supporting them because they are also afraid to disclose their HIV status by mistake or unintentionally (Mashile & Maake 2024), and thus, adolescents who do not know their condition have no reason to stick to their treatment plan. It has been demonstrated that peer acceptance and family support improve motivation and emotional stability. These findings support research showing that adolescents participating in family-inclusive adherence programmes or peer-support adherence clubs had greatly enhanced treatment continuity and psychological resilience (Kaseka et al. 2024; Mukuku et al. 2025).

Although self-motivation and ability assessment are positive reinforcements, in this study, adolescents stated that their HIV status led to different misunderstood feelings, while others claimed that it had an impact on them individually, in school, and with their students and friends. They have lost self-confidence and are not self-motivated because they are already low in self-esteem. However, they feel better when they understand their diagnosis of HIV-positive status and are aware of their HIV treatment. Adolescents living with HIV experience lower health outcomes than adults (Kose et al. 2024). Specifically in the context of self-motivation and capabilities, adolescents who understand HIV diagnosis and recognise ARV treatment are actively engaged in targeted behaviour and are influenced by beliefs about their abilities (self-efficacy) and potential outcomes rather than passively driven by external forces (Huang et al. 2024).

Within the Action States (Habits) domain of SAT, this study found that adolescents who understood their HIV status were more likely to accept their diagnosis and take responsibility for their treatment, leading to routine medication. Weber (2023) recognises that individuals’ actions are always driven by emotional impulse, but can also be rooted in established customs, habits or social norms.

Habits are a way to understand and explain human behaviour, particularly in situations where actions are performed out of a sense of duty or routine, rather than conscious deliberation. Habits have been studied in relation to a wide range of behaviours, including routine lifelong medication. These established habits enabled adolescents to maintain adherence and achieve viral suppression even when facing contextual challenges. Similarly, Gardner and Lally (2023) emphasise that habitual behaviours such as lifelong medication use emerge through repetition, environmental cues and consistent reinforcement. Their findings support the idea that adherence becomes more stable once treatment routines are embedded into daily life.

This study found that adolescents were generally optimistic about their future and expressed strong motivation to stay healthy. Their acceptance of their HIV diagnosis, willingness to take responsibility for their treatment, and establishment of regular medication routines contributed to improved adherence and sustained VL suppression. This aligns with Siripurapu and Ota (2023), who note that consistent adherence leading to viral suppression significantly reduces the risk of opportunistic infections and HIV-related mortality. Adolescents in this study also emphasised the importance of attending healthcare appointments, explaining that they wanted to avoid becoming ill or dying because of poor adherence. These findings suggest that when adolescents accept their HIV status and internalise the value of lifelong treatment, they are more likely to remain engaged in care and maintain the routine behaviours required for sustained health.

### Strengths

One of the key strengths of the study is that the data collected from various public health facilities enable a rich and diverse range of views from ALHIV. Although qualitative research does not aim to generalise statistically, this sample provides a meaningful overview of the experience of ALHIV receiving ART in a routine public sector environment. The study also adhered to established trustworthiness measures such as credibility, dependability, transferability and confirmability through continuous engagement, audit trail, high-quality recordings and systematic documentation of the research process. These strategies strengthened the rigorousness and reliability of the findings.

### Limitations

Despite these strengths, some limitations must be recognised. Firstly, HIV is a sensitive subject, and participants may have experienced feelings of pain and stigma in the interview, which may have influenced the openness of their responses. This can introduce social desirability bias, rather than recall bias. Because the study largely avoided questions requiring long-term memory, recall bias is unlikely to pose a major threat, and the assertion that they were a limitation contradicts the section on trustworthiness. Therefore, the limitations lie more in the sensitivity of the subject and in the possible restriction of disclosure. Secondly, the study was based on self-reported experiences and may not always reflect the actual behaviour of adolescents who adhered. Thirdly, the inclusion criteria are particularly focused on adolescents with an unsuppressed VL who know their HIV status. Although this is not a methodological limitation, this limits the scope of the findings because the experience may vary for adolescents who are virologically suppressed.

## Conclusion

This study used the SAT to investigate the psychosocial factors impacting VL suppression among ALHIV in Mpumalanga province, South Africa. The results show that unsuppressed VL is the product of dynamic interactions between contextual, social and personal factors rather than just a biological factor. Adherence was weakened by stigma, emotional discomfort and inconsistent caregiving, whereas VL suppression was encouraged by self-awareness, family and peer support, and consistent treatment. The study identified the adolescents’ reasons for visiting the healthcare facilities and expressed the importance of adherence to treatment. Overall, SAT provides a valuable framework for understanding the complex interactions between social, psychological and contextual factors affecting HIV-affected adolescents’ health and well-being. By including these factors in interventions, the authors can better support these young people in managing their health, reducing risk behaviours and living a productive life.
